# Stronger Neural Modulation by Visual Motion Intensity in Autism Spectrum Disorders

**DOI:** 10.1371/journal.pone.0132531

**Published:** 2015-07-06

**Authors:** Ina Peiker, Till R. Schneider, Elizabeth Milne, Daniel Schöttle, Kai Vogeley, Alexander Münchau, Odette Schunke, Markus Siegel, Andreas K. Engel, Nicole David

**Affiliations:** 1 Department of Neurophysiology and Pathophysiology, University Medical Center Hamburg-Eppendorf, Hamburg, Germany; 2 Clinical Psychology Unit, University of Sheffield, Sheffield, South Yorkshire, United Kingdom; 3 Department of Psychiatry and Psychotherapy, University Medical Center Hamburg-Eppendorf, Hamburg, Germany; 4 Department of Psychiatry, University of Cologne, Cologne, Germany; 5 Institute of Neuroscience and Medicine–Cognitive Neurology Section (INM3), Research Center Juelich, Juelich, Germany; 6 Department of Pediatric and Adult Movement Disorders and Neuropsychiatry, Institute of Neurogenetics, University of Lübeck, Lübeck, Germany; 7 Department of Neurology, University Medical Center Hamburg-Eppendorf, Hamburg, Germany; 8 Centre for Integrative Neuroscience and MEG Center, University of Tübingen, Tübingen, Germany; Harvard Medical School, UNITED STATES

## Abstract

Theories of autism spectrum disorders (ASD) have focused on altered perceptual integration of sensory features as a possible core deficit. Yet, there is little understanding of the neuronal processing of elementary sensory features in ASD. For typically developed individuals, we previously established a direct link between frequency-specific neural activity and the intensity of a specific sensory feature: Gamma-band activity in the visual cortex increased approximately linearly with the strength of visual motion. Using magnetoencephalography (MEG), we investigated whether in individuals with ASD neural activity reflect the coherence, and thus intensity, of visual motion in a similar fashion. Thirteen adult participants with ASD and 14 control participants performed a motion direction discrimination task with increasing levels of motion coherence. A polynomial regression analysis revealed that gamma-band power increased significantly stronger with motion coherence in ASD compared to controls, suggesting excessive visual activation with increasing stimulus intensity originating from motion-responsive visual areas V3, V6 and hMT/V5. Enhanced neural responses with increasing stimulus intensity suggest an enhanced response gain in ASD. Response gain is controlled by excitatory-inhibitory interactions, which also drive high-frequency oscillations in the gamma-band. Thus, our data suggest that a disturbed excitatory-inhibitory balance underlies enhanced neural responses to coherent motion in ASD.

## Introduction

Abnormalities in sensory processing and visual attention are characteristic features of ASD [1,2], the importance of which is illustrated by the recent introduction as a diagnostic criterion for ASD in DSM-5 [3]. This has led to a long tradition of research addressing perceptual abnormalities in ASD such as difficulties in the integration of visual details into a coherent percept. Perceptual integration can be investigated using dynamic stimuli, which represent the visual world more realistically than static pictures. Therefore, many studies used a coherent motion detection task, in which populations of small elements (e.g., dots) move more or less coherently (e.g., to 0, 50 or 100%) in one direction. In order to figure out the direction of the coherently moving dots, the observer needs to integrate local motion signals across space and time. Currently, empirical results on this task are highly inconsistent. Individuals with ASD performed either worse (e.g., [4–6]) or similar (e.g., [7–10]) compared to healthy controls. Moreover, in a similar task with gratings, persons with ASD were better at discriminating motion direction than a control group [11].

In addition to inconsistent behavioral results, there is little understanding of the neural mechanisms that underlie visual motion processing in ASD in the first place. Pathological processes have been suspected in the magnocellular pathway (e.g., [4,6]) or in the balance of excitatory and inhibitory neural mechanisms ([11], see also [12]). Sutherland and Crewther [13] examined motion coherence perception using EEG and measured visually evoked-potentials in individuals with high scores on scales measuring autistic traits without formal diagnosis of ASD. They found a delay in the peak of the signal component that reflected magnocellular activity. Greimel et al. [10] reported the amplitude of the N200 EEG-component, which has previously been implicated in dorsal pathway processing, as being reduced in ASD. Although both studies interpreted their findings as evidence for a dorsal stream deficiency in ASD, they did not systematically investigate a modulation of neural activity by motion coherence.

Synchronized gamma-band oscillations are a generic signature of local processing in sensory, associative and motor cortices. [14–17]. In sensory cortices gamma-band activity increases monotonically with the strength of sensory inputs [18–20] and is tuned to sensory features [21–24]. Converging theoretical and empirical evidence suggests that local gamma-band activity is driven by local excitatory-inhibitory loops for which the time-constant of inhibitory GABA-A receptors [16,25–29] and excitatory receptors [30] are shaping the frequency of gamma oscillations. Thus, gamma-band activity provides an index of local processing involving excitatory-inhibitory interactions. Moreover, gamma-band activity has been implicated specifically in feed-forward processing [17,31,32], which has also been found to be atypical in ASD [33,34]. Previous studies showed altered gamma-band responses in relation to the perception of stationary visual stimuli in ASD [35,36], but the relation between gamma-band activity and the strength of a specific sensory feature has not been systematically investigated in ASD yet.

To investigate how the autistic brain encodes the strength of visual motion signals, we based the present study on our previous work in typically developed individuals without ASD [20]. In this previous magnetoencephalography (MEG) study, polynomial regression analyses showed that frequency-specific neural activity in the dorsal visual stream was systematically related to the intensity of visual motion signals. More specifically, neural activity in the high gamma-band (60–100 Hz) increased approximately linearly with motion coherence, suggesting a functional role of gamma-band activity for motion encoding. Here, using the same parametric stimulus design, MEG methods and source reconstruction techniques as Siegel et al. [20], we addressed the questions (i) whether high-frequency population activity (e.g., 60–100 Hz) increases similarly with motion signal intensity in ASD, and (ii) whether such modulation of neural activity resides in the same cortical areas in participants with ASD compared to those without.

## Materials and Methods

### Participants

Thirteen right-handed adults with ASD and 14 right-handed, healthy controls participated in the experiments. The ASD and control groups did not differ significantly from each other with respect to age, gender or intelligence quotient (Table 1).

**Table 1:** Sample characteristics.

	ASD	Control	
	n = 13	n = 14	Statistical comparison
	mean (range)	mean (range)	
gender (female:male)	6:7	8:6	χ^2^ = .03, p = .86
mean age (years)	32 (24–45)	32.1 (23–46)	F(1,25) = .03, p = .96
verbal intelligence quotient	110.4	109.2	F(1,25) = .05, p = .82
	(92–130)	(95–143)	
Autism Spectrum Quotient	39.4 (24–48)	14.4 (9–21)	F(1,25) = 175.95, p < .001

Verbal intelligence quotient has been estimated using the German verbal „Mehrfach-Wortschatz-Intelligenz-Test” [37]; Autistic traits have been screened with the Autism Spectrum Quotient [38]: a (raw) score of ≥ 32 indicates the probability of an ASD.

n = number of participants

Participants with ASD were recruited from the outpatient clinic at the Department of Psychiatry and Psychotherapy of the University Hospital Cologne. At the Department of Psychiatry in Cologne, diagnoses were determined by two independent clinical experts following a two-step procedure. This procedure began with a first interview after referral of the patient from a practicing psychiatrist or neurologist. In cases where this first interview supported a diagnosis of ASD, participants underwent a detailed neuropsychological assessment. Next, in a second interview, the diagnosis was confirmed or rejected by a second psychiatrist (author K.V.) under consideration of the ICD-10 criteria and the neuropsychological profile. We included participants with the diagnostic categories F84.0 (Childhood autism) and F84.5 (Asperger syndrome). These participants then underwent an additional interview guided by a third independent physician (author D.S.) at the Department of Psychiatry of the University Medical Center Hamburg-Eppendorf. All participants fulfilled the cut-off for ASD according to the Autism Spectrum Quotient [38]. As expected, the Autism Spectrum Quotient was significantly higher for the ASD than the control group (Table 1).

Depression is a common comorbidity condition in ASD [39]. Two of the participants with ASD received antidepressants (one Venlafaxine and the other Fluoxetine). However, the behavioral (motion coherence threshold) and neural data (linear coefficients of gamma-band modulation by motion coherence) in these patients did not differ from the other individuals in the ASD group.

Neurophysiological and neuropsychological data (e.g., intelligence quotient, Autism Spectrum Quotient; Table 1) were obtained at the University Medical Center Hamburg-Eppendorf on the same day; structural MRIs were acquired on the subsequent day. All participants gave written fully informed consent and were paid for their participation. The study was carried out in accordance with the Declaration of Helsinki and approved by the ethics committee of the Hamburg Medical Association.

### Stimuli and Tasks

**Fig 1:**
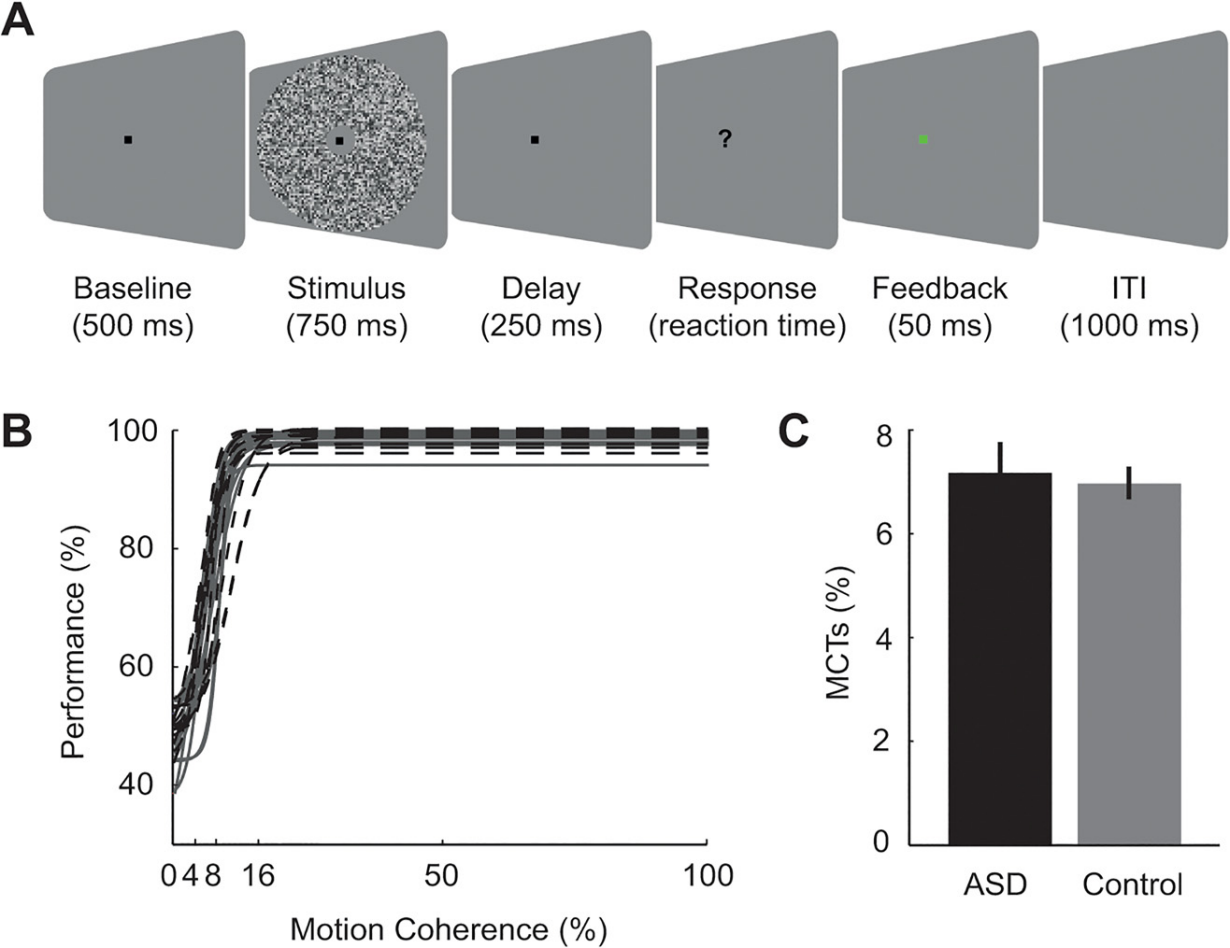
Experimental design and psychophysics. (A) Motion discrimination task. Trials started with the onset of a central fixation dot. After 500 ms, the motion stimulus was presented for 750 ms in a circular aperture. Motion coherence across stimuli ranged from 0% to 100% with either upward or downward motion direction. Following a fixed delay period, the fixation dot was extinguished and a question mark prompted the participants to report the perceived motion direction (left button = “upwards”, right button = “downwards). Participants were given a brief visual feedback (green = “correct”; red = “incorrect”). The trial ended with a blank inter-trial interval (ITI). (B) Curves represent the group-average logistic function fitted to the average motion detection performance of the control (gray solid lines) and ASD (black dashed lines) group. (C) Bar graphs illustrate the mean motion coherence thresholds (MCTs; i.e., coherence level at which 75% of motion discriminations were correct) for each group, assessed on the basis of logistic functions fitted to the individual data. Error bars represent the standard error of the mean.

Global motion perception in ASD was tested with a coarse visual motion direction discrimination task. Each motion stimulus consisted of a weighted average of a signal and a noise component. Both components consisted of normally distributed and spatiotemporally bandpass-filtered luminance noise. The mean of the luminance noise distribution was identical to the luminance of the uniform background gray. The complete black-white dynamic range of the employed video projector was spanned by +/-3 standard deviations of the luminance distribution in each stimulus. The luminance noise was spatiotemporally bandpass-filtered by multiplication in the frequency domain such that each stimulus frame contained spatial frequencies of 1.33–2.66 cycles/deg and that the frame sequence contained motion speeds of 2.4–3.0 deg/s. Each signal component consisted of only upward or downward motion. Each noise component consisted of motion in all directions. The motion coherence of each individual stimulus was set by adjusting the ratio of a signal and noise component, with 0% and 100% motion coherence corresponding to only the noise or signal component, respectively. Stimuli were presented centrally in a circular aperture (diameter: 27 deg). The stimulus was masked with the background color around the fixation dot (dot diameter 0.36 deg, mask diameter 3 deg), to rule out any stimulus interactions with the central fixation dot and to encourage monitoring of the entire stimulus field (see Fig 1A for a schematic stimulus display). Stimuli were constructed off-line using MATLAB (MathWorks Inc., Natick, MA) and presented with the software “Presentation” (Neurobehavioral systems, Albany, CA).

Each trial started with onset of a central fixation dot. After a 500 ms delay, a motion stimulus was presented centrally for 750 ms (Fig 1A). After another 250 ms delay the fixation dot was switched off, which served as the go-cue for the participants to indicate the perceived motion direction by pressing one of two designated keys (two-alternative forced-choice task: “upwards” vs. “downwards”). The participants’ response was followed by presentation of a brief square signaling the correctness of the response (green for “correct”, red for “incorrect”). This feedback served to motivate the participants and to counteract a potential response bias. Every 48 trials, participants were given the opportunity for self-paced rest. Participants performed a total of 576 trials across six levels of motion coherence: 0, 4, 8, 16, 50, and 100%. The stimulus design was fully balanced and pseudo-randomized for motion coherence and motion direction.

The dependent variable was each participant’s motion coherence threshold, that is, the minimum level of coherence at which participants performed 75% correct motion discrimination. These were determined by fitting a logistic function to each participant’s performance with the motion coherence (Fig 1B). Individual motion coherence thresholds were then submitted to a two-sample t-test to test for group differences (at p < .05 two-tailed; Fig 1C).

### Data acquisition and preprocessing

#### Data acquisition

MEG data were continuously recorded with a 275-channel whole-head MEG system (Omega 2000, CTF Systems Inc., Port Coquitlam, Canada). Participants were seated in a chair positioned in a magnetically shielded and sound attenuated room. The electrooculogram (EOG) and electrocardiogram were recorded simultaneously for offline artifact rejection and to check for fixation maintenance. The head movement in relation to the MEG sensors was also continuously recorded using head localization coils at the nasion and both ears. The participants’ maximal head movement during the first session was on average 8.9 mm for the control group and 11.1 mm for the ASD group (t(25) = -1.25, p = .23 n.s.). On the second session, control participants moved on average 10.7 mm and ASD participants 12.4 mm (t(25) = -1.03, p = .31, n.s.). The MEG data were digitized at 1,200 Hz with a low-pass filter at 300 Hz.

#### Preprocessing

All MEG data processing and analyses were performed offline using MATLAB (MathWorks, Natick, MA) and the “FieldTrip” open source toolbox (http://www.ru.nl/fcdonders/fieldtrip; [40]). The continuous data were epoched into segments of 1500 ms (starting 500 ms before visual stimulus onset), time-locked to stimulus onset (0 ms) and sorted according to experimental conditions. We reasoned that errors did not only result from motion weakness, but also from lapses of attention and/or alertness. Thus, to maximize similarity between conditions we restricted the analysis to correct trials (0%: 44 trials for the ASD group, 43 trials for the control group; 4%: 52, 51, 8%: 70, 71, 16%: 83, 87, 50%: 85, 88, 100%: 85, 89). For 0% motion coherence we randomly selected half of the trials as ‘correct’. Trials containing artifacts, which exceeded an amplitude threshold (i.e. range > 0.75 * 10^−11^ T), were rejected. Strong muscle artifacts or signal jumps were automatically detected and rejected from further analysis after visual inspection using FieldTrip functions. Data were band-pass filtered offline (0.5–170 Hz, Butterworth filter, low-pass filter order 4, high-pass filter order 3) and line noise was removed using a band-stop filter (at 49.5–50.5, 99.5–100.5, 149.5–150.5 Hz, Butterworth filter, order 4). After filtering, an independent component analysis (ICA) was applied to the epoched data, using the extended infomax ICA algorithm [41]. To reduce the noise level, we applied a principal component analysis (PCA) dimension reduction before the ICA, reducing the data to 64 independent components [42]. The number of components was chosen heuristically based on our experience to facilitate the detection of components containing artifacts. Such components representing artifacts related to eye blinks, eye movements, electrocardiographic activity and muscle activity were removed. After ICA artifact correction, the sampling rate was reduced to 400 Hz.

In order to exclude that groups differed in their fixation accuracy, EOG artifacts were identified in a semi-automatic procedure (before ICA artifact correction). The EOG channels were first bandpass filtered (1–15 Hz), then the Hilbert amplitude was calculated and finally each channel was normalized by calculating the z-score over all datapoints. The threshold was set at a z-score of 3 (corresponding to 3 standard deviations). Events with values exceeding this threshold were visually classified as saccade on the basis of their respective characteristic appearances in the EOG trace.

#### Spectral analysis

**Fig 2:**
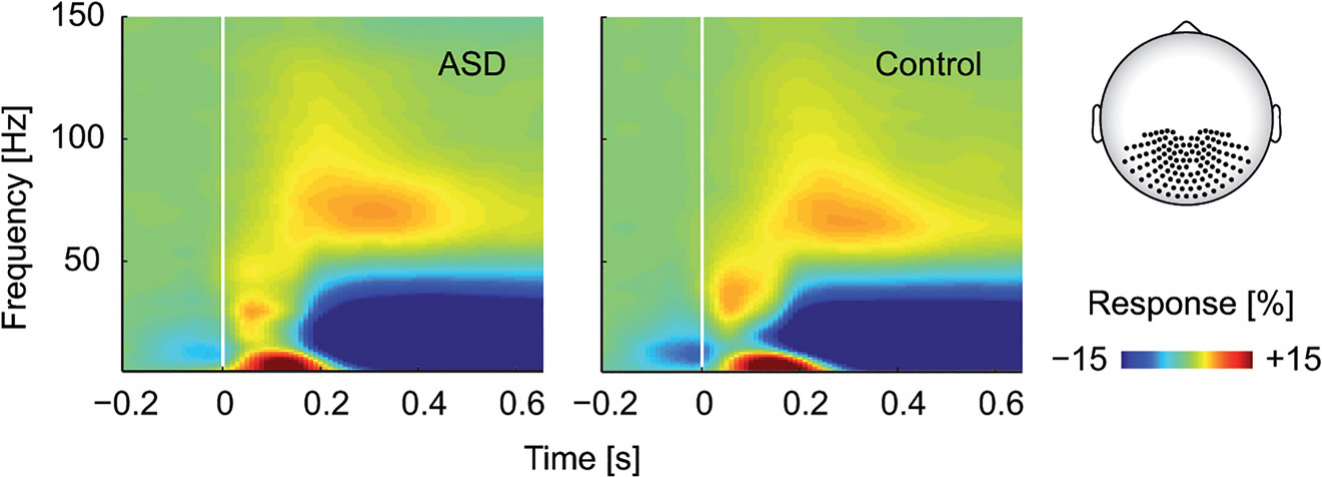
Time-frequency representation. The MEG signal was averaged across posterior sensors (highlighted on the schematic head) and all levels of motion coherence, for both groups (left: ASD, right: Control). All responses were quantified as the percentage of change in signal amplitude relative to a blank prestimulus baseline interval (400 ms before up to stimulus onset).

Data were transformed from axial to planar gradient configuration. Therefore, the planar gradient at a given sensor location were approximated by comparing the field at that sensor with its neighbors. Two orthogonal gradients in both the horizontal and the vertical direction were computed separately and then combined. After that, all spectral estimates were computed using the multi-taper method [43] based on discrete prolate spheroidal (Slepian) sequences. We computed spectral estimates across equally scaled frequencies *f* from 5 to 150 Hz (in 5 Hz steps) and time *t* from -400 to 650 ms (in 50 ms steps). A sliding window method was used with fixed taper length (200 ms) and fixed frequency smoothing (± 10 Hz). All transformations to the frequency domain were performed on the single trial level prior to averaging across trials. Thus, spectral estimates contained signal components phase-locked and non-phase-locked to the stimulus onset. The resulting total power is reported (e.g., in Fig 2) as percentage of change at frequency *f* relative to the pre-stimulus baseline (400 ms before up to stimulus onset) according to:

$$P_{poststimulus}(t,f)=100\cdot (P_{poststimulus}(t,f)-P_{prestimulus}(f))/P_{prestimulus}(f)$$

Thus, the average baseline power was first subtracted from the power of the poststimulus interval and the difference was then divided by the average baseline power.

#### Analysis of response modulation

To calculate the modulation of signal power by visual motion strength, we used polynomial regression analyses [20,44,45]. The signal *y* was modeled as a linear combination of basis functions of the stimulus variable *c* (motion coherence) equivalent to a polynomial expansion:

$$y=p_{0}+p_{1C^{1}}+p_{2C^{2}}+\ldots +p_{nC^{n}},$$

with *p* as polynomial coefficients. To independently assess the amount of accounted variance for each order of *c*, we orthogonalized the different regressors of *c*. Polynomial coefficients were estimated separately from 40 to 150 Hz (in steps of 5 Hz), from 100 up to 650 ms (in steps of 50 ms) after stimulus onset and for 117 posterior channels (Fig 2).

We tested polynomial coefficients (i) for statistical significance (against zero) separately for each group and (ii) compared them between the groups; both analyses were calculated by a cluster-based permutation test [46]. In short, all coefficients were compared between groups (or against zero) in an independent t-test. Only those coefficients exceeding a criterion (*p* < .05) were included in a clustering procedure. Clusters are defined as significant coefficients neighbored in space, frequency and/or time. On the cluster-level, a statistic was calculated by summing up the t-values within a cluster. The largest cluster-level statistic was compared with a permutation distribution under the null-hypothesis that experimental conditions (here groups) are exchangeable. Therefore, coefficients were randomly exchanged between groups and clustered in the same way like the original data. This was done for 5,000 repetitions to construct a distribution. Due to the decision on cluster-level, only one statistical comparison was performed and no further correction for multiple comparisons was necessary. We repeated our analysis with different initial thresholds (p = .1 and p = .01) and another clustering approach based on a weighted cluster mass ([47]; weight = 1), which favors peaked instead of large clusters.

To assess whether the neural modulation was related to behavioral performance, we calculated a correlation of coefficients with the individual motion coherence thresholds separately for each group. For these purposes, coefficients of the significant cluster (test against zero) were averaged across space, time and frequency.

#### Source analysis

To estimate neural activity at the cortical source level, we used the "beamforming" adaptive linear spatial filtering technique [48,49]. This source reconstruction technique uses an adaptive spatial filter, which passes activity from one specific location of interest with unit gain and maximally suppresses other activity. As linear beamforming is based on the calculation of the co-variance matrix between single channels over trials, this approach is in particular suitable for the analysis of total power. Recent studies have successfully applied linear beamforming for reconstructing the sources of frequency-specific activity in MEG [50,51].

For each participant and recording session, the co-variance matrix of 60, 70 and 80 Hz was computed independently for the stimulus (150±100 ms, 250±100 ms, 350±100 ms) and baseline (150±100 ms before stimulus onset) period using multitaper spectral estimates with ±10 Hz spectral smoothing and 3 Slepian tapers. The leadfield matrix, i.e. the physical relation between sensors and sources, was then computed using individual head models constructed from structural MRIs. The adaptive filters could induce spurious effects when comparing conditions. To avoid this, we multiplied the frequency domain data with spatial filters that were derived from the data of all motion coherence conditions.

Structural T1-weighted magnetization prepared gradient-echo images (TR = 2300 ms, TE = 2.98 ms, FoV = 256 mm, 1 mm slice thickness, TI = 1100 ms, 9° flip angle) with 1x1x1 mm^3^ voxel resolution were obtained on a 3T Siemens Magnetom Trio MRI scanner (Siemens Medical Systems, Erlangen, Germany) at the University Medical Center Hamburg-Eppendorf. For four ASD participants, structural MRIs could not be obtained at the local scanner. For those, individual MRIs previously obtained at the Department of Psychiatry and Psychotherapy in Cologne (by author K.V.) were used for source reconstruction. For one control participant without an MRI, the standard MNI brain was used. For source reconstruction, individual single shell head models with realistic shape were derived for each participant from the structural MRIs [52]. A regular 3-dimensional grid (1 cm spacing) was defined in stereotactic (i.e., MNI) space and transformed into individual head space using the individuals’ MRI. MEG sensors were aligned to the individual head geometry based on three fiducial points (nasion, left and right ear).

**Fig 3:**
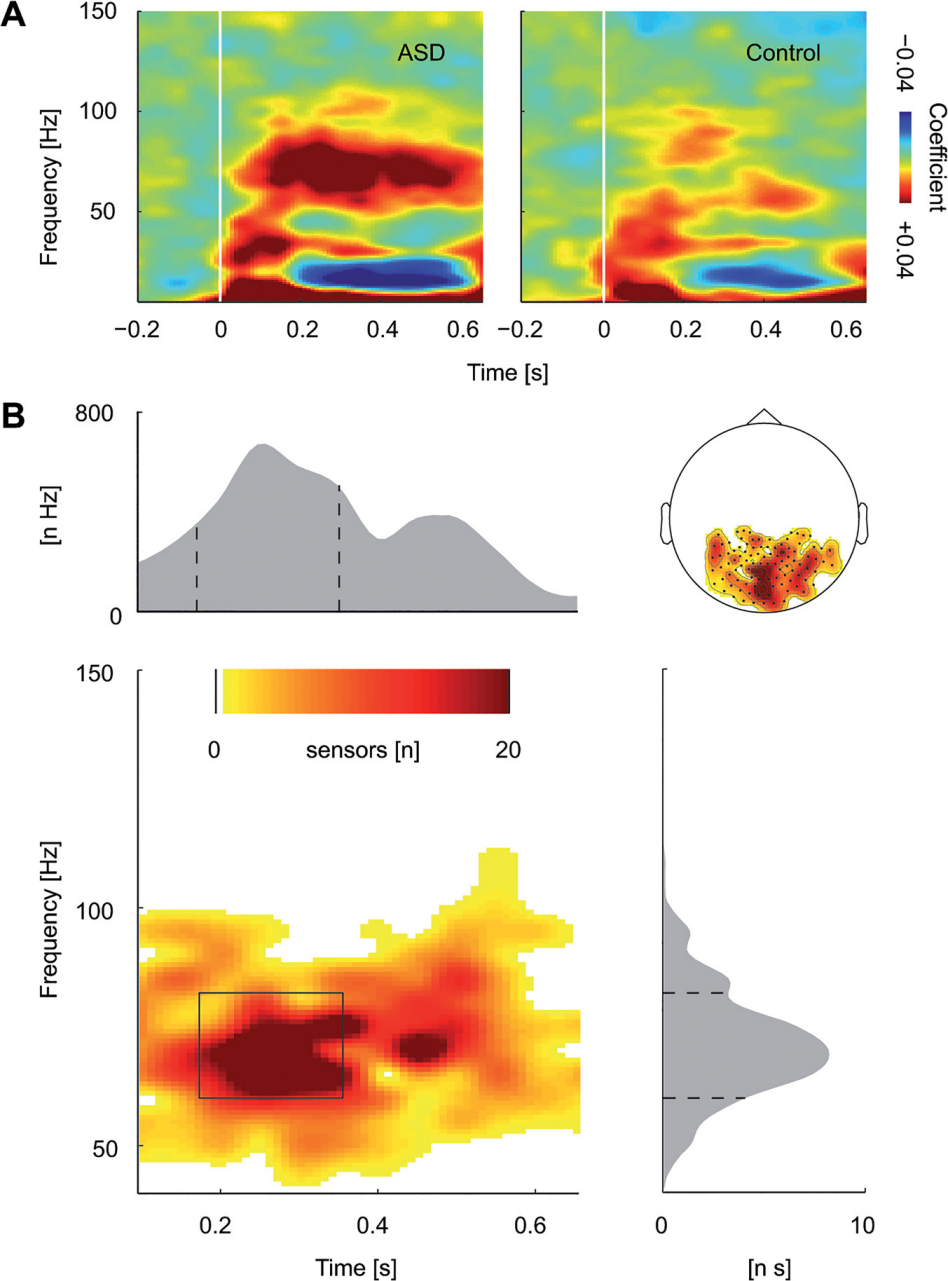
Linear coefficients estimated with polynomial regression. (A) Time-frequency representation of the linear coefficients, averaged across posterior sensors (see schematic head in Fig 2) and within groups (left: ASD, right: Control). (B) Time-frequency representation of the statistical difference of linear coefficients compared between groups (ASD—Control). Colors represent numbers of sensors belonging to the significant cluster. Y-axes of the gray-shaded curves represent the integrated number of sensors (number of sensors [n] multiplied with the respective domain, time [s] or frequency [Hz]).

After source reconstruction, data were averaged across frequencies (60–80 Hz) and time points (150–350 ms). As for all sensor-level analyses, baseline-level activity was first subtracted from activity of the stimulus interval and the difference was then divided by baseline-activity. Next, polynomial regression was applied to the response at each cortical voxel (see above). Finally, coefficients were averaged across participants. Statistical analysis was calculated at the sensor-level (Fig 3), from which our main conclusions were inferred. At the cortical source-level, we sought to only descriptively investigate the likely sources of the group effect, but not to statistically infer conclusions.

## Results

### Motion coherence thresholds

Control participants achieved 75% accuracy in motion direction detections when on average 7.0% (range 5.3–8.8%) of all dots moved in the same direction. Comparably, thresholds for ASD participants were at 7.2% (range 5.0–11.2%) motion coherence. Motion coherence thresholds did not differ significantly between the groups (*t*(25) = 0.31, *p* = .76, n.s.; Fig 1B and 1C).

### Eye Movements

The ASD group did not differ significantly from the control group with respect to the number of trials, in which saccades were made (*F*(1,25) = 0.01, *p* = .91). Moreover, the number trials containing saccades did not depend on motion coherence (*F*(3.5,88) = 1.07, *p* = .37), nor on the interaction of motion coherence by group (*F*(3.5,88) = 0.75, *p* = .55).

### Neural responses to visual motion

For both groups (ASD and control), time-frequency representations of neural responses at posterior sensors showed a profile that is typical for visual motion stimuli (e.g., [20]) (Fig 2). After stimulus onset, a transient response from about 50 to 200 ms at frequencies below 50 Hz preceded a tonic response. This tonic response consisted of an increase in signal amplitude in the high-gamma-frequency-range (>50 Hz) and a decrease at frequencies below 30 Hz. Although participants with ASD seemed to have a slightly stronger response in the high gamma-band, statistical tests revealed no significant group difference (*p*>.05).

### Modulation by visual motion strength

In our main analysis we focused on the modulation of brain activity by visual motion strength since we expected that modulation is different in persons with ASD compared to controls. We quantified the modulation of neural activity by visual motion strength using sequential polynomial regression (Fig 3A).

At first, polynomial coefficients were tested for significance separately for each group. In both groups, a significant cluster of first-order coefficients indicated a linear relationship between motion coherence and gamma-band activity (S1 Fig). The clusters extended the entire time window (100–650 ms) from 40 to 100 Hz in the control group and from 40 to 120 Hz in the ASD group. As the statistical test of second-order (quadratic) coefficients did not yield a significant cluster in either of the groups, we did not continue statistical testing of higher-order coefficients.

Correlations of linear coefficients of significant clusters (S1 Fig) with motion coherence thresholds were not significant in either of the groups (p>.05).

Comparing polynomial coefficients between groups, we found a stronger positive linear modulation for the ASD group compared to the control group. The significant cluster (p = .02) extended from 100 to 650 ms after stimulus onset and from 50 to 95 Hz (Fig 3B). All higher-order coefficients (i.e., quadratic, cubic etc.) were not significantly different between groups (p>.05). We yielded comparable results, when repeating analyses at different initial thresholds and with another clustering approach based on a weighted cluster mass [47].

### Cortical sources

**Fig 4:**
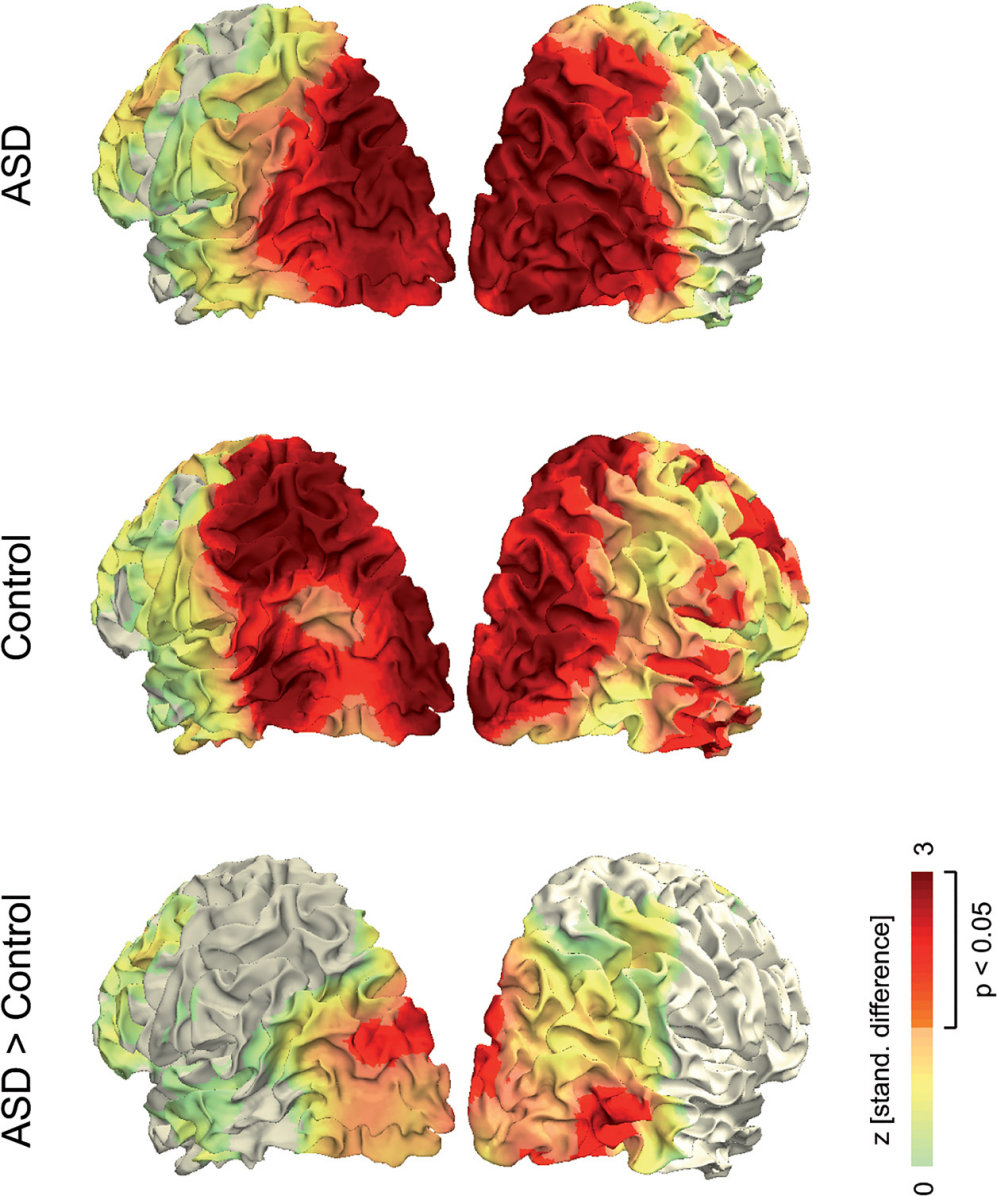
Functional overlays of linear modulation by visual motion strength. Data were averaged across time points (150–350 ms after stimulus onset) and frequencies (60–80 Hz). Projection on a standard MNI cortical surface was performed by weighting the distance between every voxel in the functional data to each surface point on the cortex. Colors represent positive z-values, while negative z-values are not shown (gray color).

Regression analyses on source level yielded functional maps that depict the cortical distribution of the positive linear modulation of gamma-band activity by visual motion (Fig 4). In both groups, this modulation was primarily located in striate and extrastriate visual areas, with a more widespread cortical distribution in the control group. The standardized difference (z-values, uncorrected) between groups suggests that the enhanced stimulus-related gamma-band modulation in ASD originated specifically from extrastriate brain areas. The location of the effect was anatomically compatible with previously reported activation for coherent motion in visual areas V3/ V6 (here in both hemispheres) and hMT/V5 (here in the right hemisphere; Fig 4) [53].

## Discussion

Here, we investigated, whether the same stimulus-response functions underlie neural processing of visual motion in ASD compared to typically developed individuals. More specifically, we addressed the questions (i) whether high-frequency population activity is similarly increased with motion signal intensity in participants with ASD compared to those without, and (ii) whether such modulation of neural activity resides in the same cortical areas. Indeed, we found significant differences in the stimulus-response functions between groups. Motion coherence predicted gamma-band power in a linear relationship that was significantly stronger in ASD than in controls. This enhanced gamma-band modulation in ASD originated specifically from motion-responsive visual areas.

### Stronger neural modulation by stimulus intensity in ASD

To determine whether a neural parameter is monotonically related to the intensity of a stimulus feature is fundamental for understanding the functional role of neural activity in sensory encoding. For example, by relating systematically increasing motion coherence parametrically to neural activity, we previously revealed a linear increase of gamma-band activity with visual motion strength in typically developed persons [20], suggesting a functional role of gamma-band activity for encoding visual motion signals. Here, we replicated this principal relationship in both, individuals without and with ASD, as in the previous study we found a linear modulation of gamma-band activity by visual motion strength. However, participants with ASD showed a significantly stronger gamma-band modulation by visual motion strength compared to control participants. Presumably, this difference arose specifically from motion-responsive visual areas V3 [54], V6 [55] and hMT/V5 [45,56].

Our findings suggest that coherent visual motion is processed differently in ASD. This is in accordance with functional imaging studies, which previously revealed generally enhanced cortical activity in visual brain areas in ASD during visual processing, for example, during face or object perception (for a review see [57]). Additionally, neurophysiological studies using EEG and MEG previously linked atypical visual processing in ASD to gamma-band activity. For example, in a study by Sun et al. [36] MEG data were recorded from participants with ASD during the presentation of Mooney faces and non-figural control stimuli. The authors reported a significant interaction of group by condition, characterized by enhanced activity in posterior regions in ASD. This result implies stronger activation of posterior brain networks in ASD associated with figural versus non-figural perception, which is in line with the present data of enhanced occipital gamma-band activity during coherent versus non-coherent motion perception in ASD. Similar studies compared the perception of illusory (e.g., Kanisza figures) and non-illusory control stimuli and found reduced differentiation by gamma-band activity of stimulus condition (illusory vs. non-illusory) in ASD ([58,59]; but see [35]). This is in contrast to our data, which rather imply an enhanced differentiation between different motion intensities in ASD. However, a direct comparison is difficult since the former studies used stationary stimuli. Moreover, these studies only contrasted two stimulus conditions, which provide limited access to the relation between stimulus features and neural responses. To study this relation, it is useful to vary stimulus features in a parametric way.

Only few fMRI or M/EEG studies systematically varied dynamic visual stimuli in a motion coherence task and compared ASD to control participants [8,10,60]. Similar to the present study, Brieber et al. [8] performed a regression analysis of the BOLD signal in relation to motion coherence. The authors did not find a significant modulation neither in area hMT/V5 nor in the whole-brain analysis, although modulations by motion coherence have previously been shown in area hMT/V5 in fMRI studies with typically developed individuals [45,56]. Nevertheless, using analyses of variance, these studies showed a main effect of motion coherence in bilateral hMT/V5 [8,60,61] and on the amplitude and latency of the P400 ERP-component at posterior sites [10]. In line with our results, the most recent study by Robertson et al. [61] found a sharper rise of BOLD activity in hMT/V5 with motion coherence in ASD, which might reflect a disturbance in local neural processing.

Since eye-movements, especially micro-saccades, could affect gamma-band activity [62], we want to rule out the possibility that our results were biased by differences in oculomotor behavior. We monitored eye movements with EOG, which did not reveal any evidence that the difference in gamma-band modulation between the ASD and control group could be due to a difference in visual fixation. Moreover, it seems very unlikely that our results were driven by electromyogenic artifacts due to micro-saccades, since both the typical MEG-topography [63] and the underlying cortical sources of micro-saccades [64] can be dissociated clearly from extrastriate visual brain areas. Yet, we cannot entirely rule out an indirect effect of micro-saccades on neurophysiological processes confounding the present results. Nevertheless, the distribution of micro-saccades is not independent from saccadic behavior, which did not differ between the groups. Thus, it seems very unlikely that our results were affected by oculomotor behavior.

### Excitatory-inhibitory imbalance and enhanced response gain

To reconcile our own and these previous findings, one may argue that we analyzed frequency-specific neural population activity, which is likely a particularly sensitive marker of the pathological circuit interactions in ASD [14,17]. In particular, converging evidence suggests that local gamma-band oscillations are driven by local interactions between GABA-ergic fast-spiking interneurons and excitatory pyramidal cells [16,25–29]. Thus, the altered gamma-band responses shown here may reflect disturbances of local excitatory-inhibitory interactions in ASD.

Disturbance of excitatory-inhibitory interactions have previously been suspected as a pathophysiological mechanism in ASD. Rubenstein and Merzenich [12] characterized brain processing in ASD by a disproportionate high level of excitation or a disproportionate weak inhibition. This notion is supported by increased prevalence of epilepsy in ASD [65] and a reduced efficacy of the GABAergic system in ASD [66–68]. The GABAergic system has not only been implicated in generating gamma oscillations, but also in controlling neural response gain (e.g., [69]). Specifically, reduced inhibition may lead to an enhanced responses gain, i.e. stronger increase of neuronal activity with stimulus intensity. In accordance with this hypothesis Foss-Feig et al. [11] found enhanced motion perception in children with ASD when high-contrast drifting gratings were presented. To explain this performance advantage in ASD, the authors proposed an abnormally enhanced response gain in ASD (see also [70]). Supporting Foss-Feig and colleagues’ behavioral findings [11], here we show an enhanced modulation of neural responses by feature intensity, which gives further evidence for an enhanced response gain in ASD.

We suggest that the stronger neural modulation in ASD arose from motion sensitive brain areas V3 [54], V6 [55] and hMT/V5 [44,55]. In area hMT/V5 cells with an excitatory center and an inhibitory surround [71,72] are thought to be crucially involved in motion perception [73,74]. This center-surround antagonism diminishes the response to large coherent stimuli [56]. If this antagonism is disturbed in ASD due to reduced inhibitory activity, this will lead to increased neural responses to coherent stimuli, i.e. an enhanced response gain in ASD, as shown here.

Regulation of sensitivity to different levels of stimulation is a common problem faced by all sensory systems [70,75]. Thus, beyond motion perception, disturbance of such control mechanisms might bear implications for a broad range of perceptual abnormalities in ASD [76–78].

### Gamma-band modulation was not linked to behavior

Notably, enhanced modulation of gamma-band activity by motion intensity was not associated with better motion discrimination performance in the present task. Furthermore, there was no significant correlation between gamma-band modulation and psychophysical performance. Together, this suggests that gamma-band activity was not the limiting neural factory for performing the present psychophysical task. This is in line with our previous study [20], in which we found little gamma-band modulation across very low motion coherence levels, for which psychophysical performance increased steeply. Conversely, as for the present data, behavioral performance was already saturated at intermediate levels of motion coherence where gamma-band activity increases reliably.

Not only local encoding of sensory information, reflected in gamma-band activity, but also integrative processes involved in accumulating sensory information across time may critically determine psychophysical performance. Indeed, Donner, Siegel, Oostenveld, Fries, Bauer and Engel [79] found fronto-parietal beta-band activity that may reflect sensory evidence accumulation to predict coherent motion detection performance. Along the same line, impaired behavioral performance in ASD has been found for long, but not for short stimulus viewing durations for stationary and dynamic stimuli ([80,81]; but see [82]). Furthermore, in a task with moving gratings, Foss-Feig et al. [11] measured shorter duration thresholds in ASD suggesting that individuals with ASD can even more quickly accumulate low-level motion information from moving stimuli. Thus, one may speculate that with longer or shorter stimulus durations participants with ASD may have performed worse or better than controls in the present task, respectively.

### Conclusion

Atypical visual processing has been suggested as a characteristic phenomenon in ASD [57,76–78], and has often been probed with tasks that require perceptual integration such as the detection of coherent motion signals from noise (e.g., [4]). Here, we found an abnormally stronger neural modulation by visual stimulus intensity in ASD, that is, increases in visual motion strength were associated with stronger increases of visual cortical gamma-band activity in ASD compared to controls. This suggests an excitatory-inhibitory imbalance that leads to an excessive sensory response gain in ASD. A disturbance of gain control in ASD might bear implications for a broad range of perceptual abnormalities in ASD.

## Supporting Information

S1 Fig Time-frequency representation of linear coefficients, statistically thresholded against zero. A: ASD, B: Control. Colors represent numbers of sensors belonging to the significant cluster.
(EPS)
