# Correction: Stronger Neural Modulation by Visual Motion Intensity in Autism Spectrum Disorders

**DOI:** 10.1371/journal.pone.0134769

**Published:** 2015-07-29

**Authors:** The PLOS ONE Staff

The Data Availability statement for this paper is incorrect. The correct statement is: Data are available from Dryad Digital Repository: http://dx.doi.org/10.5061/dryad.fc40s. The Publisher apologizes for this error.
